# Supplementary material for: Affective evaluation of images influences personality judgments through gaze perception
Source: PLoS One. 2020 Nov 5;15(11):e0241351. doi: 10.1371/journal.pone.0241351 (PMC7643958; doi:10.1371/journal.pone.0241351)
Supplement: S1 Appendix — (DOCX) [file pone.0241351.s001.docx]

S1 Appendix

*IAPS and OASIS Numbers of Used Images*

*Experiment 1*

*Positive images*: 2040 (*i*), 2070 (*i*), 2071 (*i*), 7282 (*i*), 7325 (*i*), 7430 (*i*), 7450 (*i*), 1463 (*i*), Lake 14 (*o*), Food 6 (*o*), Flowers 6 (*o*), Flowers 1 (*o*), Fireworks 3 (*o*), Cat 4 (*o*), Cat 5 (*o*), Baby 9 (*o*), Baby 5 (*o*), Rainbow 2 (*o*)

*Negative images*: 1300 (*i*), 1301 (*i*), 1321 (*i*), 1525 (*i*), 1726 (*i*), 1930 (*i*), 1051 (*i*), 9622 (*i*), Explosion 3 (*o*), Fire 11 (*o*), Lightning 2 (*o*), Tornado 1 (*o*), Snake 2 (*o*), Snake 3 (*o*), Snake 4 (*o*), Snake 5 (*o*), Snake 6 (*o*), Shooting 1 (*o*),

*Experiment 2*

*Positive images*: 2040 (*i*), 2070 (*i*), 2071 (*i*), 7282 (*i*), 7325 (*i*), 7430 (*i*), 7450 (*i*), 1463 (*i*), Lake 14 (*o*), Food 6 (*o*), Flowers 6 (*o*), Flowers 1 (*o*), Fireworks 3 (*o*), Cat 4 (*o*), Cat 5 (*o*), Baby 9 (*o*), Baby 5 (*o*), Rainbow 2 (*o*)

*Negative images*: 1300 (*i*), 1301 (*i*), 1321 (*i*), 1525 (*i*), 1726 (*i*), 1930 (*i*), 1051 (*i*), 9622 (*i*), Explosion 3 (*o*), Fire 11 (*o*), Lightning 2 (*o*), Tornado 1 (*o*), Snake 2 (*o*), Snake 3 (*o*), Snake 4 (*o*), Snake 5 (*o*), Snake 6 (*o*), Shooting 1 (*o*),

*Neutral images*: Barrels 1 (*o*), Billiards 1 (*o*), Bottle 1 (*o*), Bricks 1 (*o*), Cardboard 3 (*o*), Lake 4 (*o*), Office supplies 4 (*o*), Paperclips 2 (*o*), Path 1 (*o*), Pinecone 3 (*o*), Roofing 4 (*o*), Yarn 3 (*o*), Windmill 1 (*o*), Street 5 (*o*), Street 1 (*o*), Storage 2 (*o*), Solar panel 1 (*o*), Sidewalk 4 (*o*)

*i = IAPS numbers, o = OASIS numbers*
