# Supplementary material for: Affective evaluation of images influences personality judgments through gaze perception
Source: PLoS One. 2020 Nov 5;15(11):e0241351. doi: 10.1371/journal.pone.0241351 (PMC7643958; doi:10.1371/journal.pone.0241351)
Supplement: Attachment — Submitted filename: 190902_responses.docx [file pone.0241351.s003.docx]

September 13th, 2019

Reference: PONE-D-19-14867

Resubmission

“Affective evaluation of images influences personality judgments through gaze perception”

We appreciate for your highly valuable comments on our paper. We have made changes to our writing by following your comments. Our responses to the reviewers’ comments are below.

**RESPONSE TO REVIEWER 1:**

**Comment 1:**

*The authors should explain why they chose to use Cohen’s f for their sample size calculation, and why they chose a medium effect size. Were these choices based on previous published data?*

*I tried to replicate the sample size calculation the authors employed in E1 using G*Power, however they did not provide all the information needed to run the calculation. If I assume the statistical test is a repeated measures ANOVA, within factors, and select 1 group and 6 measurements, my result it close to the one the authors obtained (19 participants instead of 18), however it would be good if the authors could clarify their choices here. In E2, could the authors explain the rationale for why they chose a sample size much larger than the sample size returned by their effect size calculation.*

**Response 1:** We thank the reviewer for informing us about the lack of information needed to run this calculation. In accordance with this comment, we have added the information about the calculation for the sample size to Materials and methods.

Obviously, we could not know the appropriate effect size for our experiments in advance, so we used the standard setting (i.e., *Cohen’s f* of 0.25 in G*power). Finally, we investigated a relatively large number of participants than the calculated sample size after considering the possibility of missing data.

**Comment 2:**

*For the sake of completeness, the authors should provide the image numbers for all 36 images used in the study in the Appendix, instead of just a subset of the images.*

**Response 2:** We appreciate the reviewer's comment on this point.

We agree with the reviewer’s comment. Accordingly, we have included the image numbers for all images in the  *Supporting information.*

**Comment 3:**

*For the faces, how were the eyes altered to depict leftward and rightward gaze? Were the faces matched for attractiveness? Previous research shows that more attractive individuals are seen in a more positive light (e.g., Bascandziev & Harris, 2014), thus it is imperative to control for attractiveness in these types of studies.*

**Response 3:** We thank the reviewer for this comment. We created the faces with the averted gaze by cropping and changing the pupils of the faces with a directed gaze. We have included an explanation to the Stimulus and apparatus section providing more information about image processing.

As this reviewer observes, we also think that the control of attractiveness of the faces is very important in this study. In our study, the face images were randomly assigned to the conditions per participants. Therefore, we believe that the effect of facial attractiveness did not significantly influence our findings.

**Comment 4:**

*The Methods section is currently lacking information on the Apparatus used to present the computer task, along with the Design section to indicate the conditions, etc. Please provide details as to the computer, monitor, program used to present the task, etc.*

**Response 4:** Thank you for this comment. We agree with the reviewer’s comment. Accordingly, we have added information about the apparatus to Materials and methods.

**Comment 5:**

*Which keys were used to respond to the target? This should be noted in the manuscript, due to the well-known Simon effect. This effect, whereby a left key press made by a left hand to signal a left location, leads to a congruent spatial stimulus-response mapping situation, which makes it difficult to tease apart whether it is attention or motor preparation that is leading to the RT effects. As there is some evidence that social attention is affected during Simon tasks (e.g., McKee, Christie, & Klein, 2007; Zorzi et al., 2003), the authors should be mindful of the conclusions they draw depending on how the responses were provided.*

**Response 5:** We welcome this insightful comment. We should have explained the methods of our study more clearly. Therefore, we have added an explanation about response mapping to the Materials and methods.

**Comment 6:**

*Can the authors explain their choice to use median RTs instead of the more conventional mean RTs?*

**Response 6:** We wish to thank the reviewer for these comments. When we examined the distribution of the data for each participant, we found bimodality and asymmetry on the distribution of response times for each participant. Therefore, we used the median value for analysis. Several previous studies also have used median values in the analysis for the gaze cueing tasks (e.g., Bayliss, Paul, Cannon, & Tipper, 2006).

Bayliss, A. P., Paul, M. A., Cannon, P. R., & Tipper, S. P. (2006). Gaze cuing and affective judgments of objects: I like what you look at. *Psychonomic Bulletin & Review*, *13*(6), 1061–1066.

**Comment 7:**

*Figures 2 and 5. The results would be clearer if the y-axis was scaled to start around 300ms instead of zero, and go until around 400ms instead of 600ms.*

**Response 7:** We thank your comments.

To assist these interpretations for readers, we have changed the y-axis in Figure 2 and 4 (Figure 4 is new version of Figure 5).

**Comment 8:**

*page 11, line 176. How is the finding (two-way interaction) deemed marginal with a p value of 0.08, whereas on the next page, lines 188 and 189 the effects are deemed to be “no effect” with p values of 0.09 and 0.08. The authors should avoid usage of “marginal”; if using NHST, then something is either significant or not.*

**Response 8:** Your reading of our interpretations is quite accurate; we appreciate your comment on this point. We also think that we should have a consistent interpretation for the *p* values of 0.08. Because we considered that these points should be changed, we have rewritten the interpretation for the analysis in the Results.

**Comment 9:**

*line 223 – the word “thus” is repeated twice.*

**Response 9:** We thank your comments on this point. We have changed from *“thus”* to *“this”.*

**Comment 10:**

*page 16, lines 253-255: “Second, because the valid-cue faces were predictive of both the location and valence of the targets, we could not determine which type of predictability was critical for the perceived trustworthiness to be modulated.”*

*To clarify, aren’t both the valid-cue and invalid-cue faces predictive of location and valence? Specifically, the invalid-cue faces will ALWAYS look away from the target location (which needs to be implicitly learned just as in the valid-cue face condition), and certain faces predict positive images while others predict negative images, no? Thus, the difference between valid-cue and invalid-cue faces is the ‘helpfulness’ participants attribute to the gaze information (and thus the face itself). I suggest revising this sentence to incorporate this more nuanced difference.*

**Response 10:** We thank you for this insightful comment. Indeed, our writing was unclear. To help the interpretation of readers about the differences across the conditions, we have rewritten and added the explanation about these conditions to the introduction in Experiment 2.

**Comment 11:** *I appreciated that the authors collected two measures of trustworthiness for each face in E2. I also wonder, however, if the single measure of trustworthiness done in E1 could be driving the difference between ratings for positive and negative images in the valid-cue condition. One way the authors could partially mitigate this concern would be to calculate the standard deviation across the two scores per face in E2, and demonstrate that the variability is low. This would give additional weight to the conclusions drawn from E1.*

**Response 11:**

We appreciate your comment and advice. In accordance with your comments, we assessed whether there were the differences between two scores per face in Experiment 2. As a result, we did not find the differences between these scores. Therefore, we consider that the facial evaluation in Experiment 2 was not strongly influenced by the variability between the scores.

**Comment 12:**

*Figures 3 and 6. For reader ease, the authors should add the average trustworthiness rating scores for each condition above the corresponding bar.*

**Response 12:**

Thank you for your comment. In accordance with your idea, we have added the rating scores to Figures 3 and 5 (Figure 5 is new version of Figure 6).

**Comment 13:**

*I cannot find the repository where all of the raw data are available. Can the authors please provide this information.*

**Response 13:**

We thank you for your comment. We have added the requested information to the Supporting information.

**RESPONSE TO REVIEWER 2:**

**Comment 1:**

*The research question is introduced clearly and addresses an intriguing issue. Unfortunately, several limitations prevent me from supporting the publication of this study.*

*My main concerns relate to the sample and the statistical approach. The rationale beyond the power analysis to establish the sample sizes of both experiments (i.e., 20 and 24 participants, respectively) is unclear. For example, it is unclear how the reference effect size of 0.25 was established (e.g., previous research?) and whether the Authors used an a-priori or post-hoc analysis.*

**Response 1:**

We wish to express our deep appreciation to this reviewer for this insightful comment surrounding this topic. . As you wrote, the information about the sample analysis was not fully in our paper.

We conducted a-priori power estimation in our study. Moreover, because we obviously could not know the appropriate effect size for our experiments in advance, we used the standard setting (i.e., effect size (*f*) of 0.25 in G*power). In accordance with your comments, we have added the relevant information about sample analysis to the Materials and methods.

**Comment 2:**

*Additionally, given the subtle modulation that this study aims at investigating, the resulting sample sizes appear small – especially considering the relatively small number of trials that were used (i.e., 216).*

*Possibly as a result of this relative lack of power, the results are uncertain and sometimes not statistically significant for the central effects of interest. The additional Bayesian analyses are, in my opinion, insufficient to make the results more reliable – which seems the only or main reason why they were added. I agree that Bayesian analyses can be highly informative, and sometimes even preferable to the NHST approach. However, their rationale should be explained clearly, and more details should be given on how they were implemented (e.g., description of priors).*

*In terms of overall results, the only reliable finding I can appreciate is that validly cuing faces were perceived as more trustworthy than invalidly cuing faces. This finding is consistent with previous literature but is not informative to Authors’ hypotheses.*

*Thus, overall, I think that this study is not ready for publication. However, I also think that this paper pursues an interesting line of research, and I wish the Authors the best of luck with the investigation on this topic. Below are some additional comments (in no particular order) that I hope will benefit Authors’ future work.*

**Response 2:** Thank you for your insightful comments. As you wrote, we felt that we needed to ascertain if we had enough power, taking into account the number of trials (i.e., 216 trials). Therefore, we performed the power analysis by PANGEA that is web-based power application that can estimate power using information of the trials. As a result, we obtained a power of 0.911 (PANGEA default effect size [*d*]: 0.45, iterations: 36 times, participants: 20). We consider that it indicated that at least the analysis in PANGEA confirmed sufficient power.

Thank you for your advice on the lack of information on Bayesian statistics. In our study, we used the Cauchy distribution with a default scale 0.707 in JASP that is analysis software. In accordance with your comments, we have added an explanation about the role of Bayesian analysis in these Results.

Although the effect shown in our overall results may not seem large, we consider that our findings fill the certain blanks that previous studies have not considered. So, we believe that it is meaningful to report this.

**Comment 3:**

*The expression “marginally significant” is inappropriate (page 11, line 176) as it does not reflect the arbitrary nature of the p-value. Furthermore, it appears that Authors use this label arbitrarily, as for example similar results (p=.08, eta=.15) are described as “marginally significant” (page 11, line 176) for the two-way interaction of interest and considered non-significant a few lines below (page 12, line 189). Thus, the section exploring the two-way interaction should be eliminated.*

*Related to the first point, the inclusion of correlations does not seem appropriate (at least with the separation by target type) because they follow results that appear unreliable (i.e., the interaction). Additionally, the correlations are not significant; thus, it is unclear what the value of Figure 4 is.*

**Response 3:** We thank the reviewer for this comment.

We also think that these points should be corrected to accommodate the concerns of this reviewer. Accordingly, we have rewritten the interpretation for the analysis in the Results.

Moreover, we agree with your idea for Figure 4*.* Therefore, Figure 4 was removed from our paper.

**Comment 4:**

*The description of the methods of Experiment 1 is unclear, particularly on whether face validity and target valence target were manipulated independently. On first reading, I assumed that they were manipulated independently, which would be recommended. However, I reconsidered this assumption later on, when Experiment 2 was introduced. The Authors write “Second, because the valid-cue faces were predictive of both the location and valence of the targets, we could not determine which type of predictability was critical for the perceived trustworthiness to be modulated” (page 16, lines 253-255). This sentence suggests that valid-cue face always cued positive images, and this is a major issue casting doubts on the overall procedure of Experiment 1.*

**Response 4:**

We thank your insightful comments. Our writing was unclear.

In Experiment 1, each face was associated with the same gaze pattern and the same valence of the targets during the gaze-cueing tasks. Previous studies have suggested that persons with consistent attitudes and behaviors tend to be evaluated as superior in the personality and intelligence. Therefore, it remains unclear whether the consistency of the gaze and the target types was critical for the perceived trustworthiness to be modulated. So, we examined whether such consistency influenced the facial trustworthiness by manipulating the predictability of the target valence.

To help the interpretation for readers about the purpose in Experiment 2, we have added an explanation of this in the introduction in Experiment 2.

**Comment 5:**

*Experiment 2 substantially replicates the main effect of Experiment 1 and does not add much in its present form. Furthermore, the additional analyses (“Does history of target valence effect facial evaluation?” page 23, line 382) are poorly introduced/explained, and it is unclear what their contribution is, given the lack of clear and reliable results.*

**Response 5:** We thank the reviewer for this comment.

After reading comments 4 and 5, we felt that we had conveyed an unclear explanation about the purpose of Experiment 2. So, we rewrote relevant parts of the introduction of Experiment 2.

In addition, following your comments, we also thought that we should explain the additional analysis more clearly. Accordingly, we have added the explanation to the Additional analysis.
