# Supplementary material for: Affective evaluation of images influences personality judgments through gaze perception
Source: PLoS One. 2020 Nov 5;15(11):e0241351. doi: 10.1371/journal.pone.0241351 (PMC7643958; doi:10.1371/journal.pone.0241351)
Supplement: Attachment — Submitted filename: Response to Reviewers.docx [file pone.0241351.s004.docx]

December 19th, 2019

Reference: PONE-D-19-14867

Resubmission

“Affective evaluation of images influences personality judgments through gaze perception”

We appreciate for your valuable comments on our paper. We have revised the manuscript by following your comments. Our responses to the reviewers’ comments are below.

**RESPONSE TO REVIEWER 1:**

**Comment 1:**

*sample size: While the authors are correct in stating they cannot know the effect sizes for their study beforehand, they do have access to previous literature, and could thus calculate an effect size that would be more representative of standard cueing effects when manipulating trustworthiness, predictiveness and affective faces/targets. Opting for a “standard setting” is problematic when the authors don’t know why a particular program has a default listed*

**Response 1:** Thank you for the comment.

As you stated, there have been similar studies, however, since our study and the previous study did not have the same experimental design, we did not refer to the effect sizes of the previous studies.

Also, we feel that the expression "standard settings" may have caused a misunderstanding. In order to avoid large power overflows and too little power, we calculated the sample size by using the medium effect size (*f*).

**Comment 2:**

*methods details: thank you for the addition information. The manuscript continues to lack a Design section, however, which is a point raised in the first round of reviews. The Design section should list each of the factors (and their levels). It remains unclear whether the valid gaze cues were equally likely to look at positive and negative images, or whether these two points were confounded. Including details about the design within the methods section rather than as a small discussion point between the two experiments would clear up a lot of confusion. Further, it is unclear to me how the design of E1, where gaze-validity and target valence are confounded, will answer the authors main question of whether the emotional valence of a gazed object affects facial impression of the gazer, thus the rationale for including experiment 1 as it stands is unclear.*

**Response 2:**

Thank you for the comment.

We agree with the reviewer’s comment. According to the comment, we have included the details of the experimental designs in the Design section*.*

**Comment 3:**

*median RTs: the authors should include information about the distribution of the data and rationale for selecting medians in the manuscript proper.*

**Response 3:** We thank the reviewer for this comment.

We have included information about the distribution. Since it was not a normal distribution, we used the median.

**Comment 4:**

*use of the term “marginal”: the authors have failed to remove the word “marginal” from both lines 181 and 193.*

**Response 4:** Thank you for this comment. We have removed the word *“marginal”*.

**Comment 5:**

*Design details: I am still unclear about the design of the study. Initially I believed that predictiveness and target valence were fully crossed, with 4 groups of faces. One group of faces always looked toward positive targets, the second group always looked at negative targets, the third group never looked at positive targets and the last group of faces never looked at negative targets. Then, when reading the other reviewer’s comments, it became clear that there is a confound between gaze validity and target valence, which means the data are confounded, and the design is not able to address the research question. After reading the revised section between E1 and E2, it is still unclear what the authors believe the first study adds to the literature.*

**Response 5:** We welcome this insightful comment. We should have explained the design of our study more clearly. Therefore, we have included an explanation about the experimental settings in the Design section. Moreover, to assist the interpretations by the readers, we also have included a Figure in the Design section.

**Comment 6:**

*trustworthiness ratings: how did the authors assess “whether there were the differences between two scores per face in Experiment 2.”, as they state in the letter to the reviewers? I suggest reporting the corresponding stats in the letter, or in the manuscript proper.*

**Response 6:** We thank you for your comments. We have included these results in a footnote of the Results section (p.24).

**Comment 7:**

*Raw data: thank you for including the raw data. The response times appear to be coded in seconds; the authors may want to either make a note of this somewhere, or convert to milliseconds to match their graphs*

**Response 7:** We appreciate your comment on this point. We have included an explanation about the response times in S2_Appendix.

**RESPONSE TO REVIEWER 2:**

**Comment 1:**

*P.4 “For example, when we observe someone looking at a particular location, our attention automatically shifts to the same location (‘joint attention’)” – this reads like a definition of gaze-following, rather than joint attention.*

**Response 1:** Thank you for your insightful comments. We have revised this point accordingly (p.4-5).

**Comment 2:**

*P.4 “Also, the viewer’s likability of the face was rated after the response to the target” – Regarding Bayliss & Tipper (2006), participants rated the trustworthiness of faces, not likability. This is an issue throughout the results section as well, where the authors describe their participants’ ratings as likability ratings when they were trustworthiness ratings. Care should be taken to be precise, particularly because this learning effect is not present when participants are asked how much they like the faces, only how much they trust them (cf. Strachan et al., 2016).*

**Response 2:** We express our deep appreciation to this reviewer for this insightful comment. In accordance with your comments, we have revised the explanation about previous studies (p.4, line 56-59).

**Comment 3:**

*P.6 “We investigated relatively more participants compared to the calculated sample size after considering the possibility of missing data” – the authors use the same rationale in Experiment 2 to justify an even larger sample size (and a much bigger increase relative to the power calculation). I see that the authors have responded to similar comments from the other reviewers but I am still confused. If missing data are such a concern then why is no missing or excluded data reported in Experiment 1? If there were no missing data in Experiment 1, why is it such a pressing concern in Experiment 2? Any missing data must be reported and justified.*

**Comment 15:**

*P.18. See above comment re:p.6 regarding power calculations, sample size, and missing data. It is not clear what data might be missing when no filtering or exclusion criteria are reported. Please also report the reason for the increased sample size in the manuscript text.*

**Response 3 and 15:** We thank the reviewer for this comment. There were no missing data in our experiments. At times, certain participants interrupted the experiment of another study, and therefore, we attempted to recruit a slightly higher number of participants for all experiments.

Also, there was a delay in stopping the recruitment on the Internet when the required number of people were collected, which resulted in a slightly large number of participants in Experiment 2.

Our discussion is not only based on the results of ANOVAs, but also the Bayesian analysis. Moreover, the effect shown in our overall results is not large. Therefore, we believe that the number of participants in our study did not strongly influence the results of our study.

**Comment 4:**

*P.6. I am not convinced by the rationale for the correlation analyses. As I understand it, the prediction is that the participants who are more strongly affected by misleading gaze (and therefore experience greater RT costs) might be more likely to discriminate valid from invalid faces on the basis of trust. But I am not sure how this fits with the hypothesis that perception of a sender’s gaze alone would be sufficient to modulate trustworthiness without any attention reorienting. Evidence suggests that this trust learning is not driven by attention reorienting per se, but by a negative emotional reaction that is the result of misleading gaze (Manssuer et al., 2016). This emotional reaction (and the trust learning) do not appear to be related to the magnitude of the gaze-cueing cost, but by the interpretation of it.*

**Response 4:** We thank for your insightful comments. We are sorry that our writing caused a misreading. As you have mentioned, previous studies have shown that attentional orientation does not appear to be related to the results of the facial evaluation. We conducted a correlation analysis because we wanted to confirm whether our results were consistent with the results of previous studies (i.e., whether the boosted facial evaluation was explained by the observer's attentional orienting).

We have revised the explanation of the hypothesis in the introduction (p.6, line 87-92).

**Comment 5:**

*P.8-9. Details of the design are missing or incomplete. For example, “Since the pairs of a face and target were randomized for each trial” – this suggests that faces were paired equally often with any given target, and nowhere is it made explicit that faces were only paired with targets of a given valence. A dedicated paragraph on the experimental design could make this clearer. I note that another reviewer has already raised this point in a previous review (R1: Comment 4), but I still found the description unclear.*

**Comment 6:**

*I was not sure why no-cue faces that did not move their eyes were used – whether this was just as a control or an experimental condition with specific hypotheses. As I am not clear on why these faces were included in the design, this makes the findings much more complicated to interpret. Spell out clear justification, hypotheses and predictions for all conditions. This will make it much easier to see what contrasts are relevant to what.*

**Response 5 and 6:** We thank the reviewer for this comment.

According to your comments, we have more clearly explained the experimental design (including the explanation about no-cue faces in the Design sections of Experiments 1 and 2.

**Comment 7:**

*P.9 “a number from 1 to 9 appeared” should read “a scale from 1 to 9 appeared”*

**Response 7:** Thank you for your comments. We have revised this point (p.9, line 144).

**Comment 8:**

*P.9. A section in the Methods where the authors detail their data analysis plan and rationale (details of the frequentist ANOVA with post-hoc comparisons, Bayesian contrasts with clear explanation of what is tested and why, and the correlation analysis) would be helpful.*

**Comment 10:**

*P.11-12. It is quite difficult to follow so many different analyses and understand which are justified follow-ups and which are not. Breaking up this whole results section into separate paragraphs and relating each analysis back to the specific hypothesis it is testing would make it easier to see which analyses are follow-up and which are conceptually separate.*

**Response 8 and 10:** We welcome this insightful comment. As you have stated, we also felt that separating the results by type of analysis obscures the understanding of the purpose and hypothesis of each analysis. Therefore, we changed the layout of the results.

**Comment 9:**

*P.11. “Furthermore, the two-way interaction was marginally significant”. Please drop the phrase ‘marginally significant’ throughout the manuscript – I believe both reviewers of the previous version also make this point.*

**Response 9:** We thank the reviewer for this comment. We have removed this word (p. 13).

**Comment 11:**

*P.12 & P.22: “Mean of trustworthiness scores per conditions are illustrated in upper each bar.” Change to “Means of trustworthiness scores for each condition are shown above each bar”*

**Response 11:** We thank the reviewer for this comment. We have revised this point.

**Comment 12:**

*P.13. Line 214: Please always refer to a BF as either BF10 or BF01. Given that the authors talk about both in the text, it is important to be clear.*

**Response 12:** We thank the reviewer for this comment. We have added information about the Bayes factors.

**Comment 13:**

*P.13 & P.22. It is not clear if these Bayesian contrasts are directional or not. I think it is valid to form directional hypotheses on the basis of previous studies (e.g. that ratings to valid faces will be more positive than for invalid faces), but it is not clear at the moment what alternative hypothesis the authors are actually testing.*

**Response 13:** We thank the reviewer for this comment. We agree with your comments. To clarify the hypothesis, we have added information about the hypothesis of Bayesian analysis.

**Comment 14:**

*P.14-15. Reiterate the rationale for the correlation analysis and the hypotheses it is testing. It would also be useful to explain how this finding ought to be interpreted, as it does not appear in the discussion section.*

**Response 14:** We thank the reviewer for this comment. According to your comment, we have added the rationale and hypothesis for the correlation analysis.

**Comment 16:**

*P.19. The use of “non-predictive” to refer to faces that consistently provide valid or invalid cues equally often to positive and negative images is confusing considering that this is a gaze cueing paradigm. All faces in Experiment 2 are predictive if one learns the contingencies, and they are predictive of the task-relevant feature (the target location). Non-predictive faces, such as were used in the original Bayliss & Tipper design, would be faces that provide 50/50 valid and invalid cues. As the authors are referring to a different kind of contingency, I would advise using a different term throughout to avoid confusion (maybe ‘inconsistent’ in their preference for positive or negative targets?).*

**Response 16:** We appreciate the reviewer for this comment. As you have stated, we also thought that the expression “non-predictive” confounded the readers’ understanding. Therefore, we changed the name of the condition from “non-predictive” to “random”.

**Comment 17:**

*P.19. Using 4 types of faces in a within-subjects design is quite complicated for this kind of learning task, and I suspect that not only is the complicated design working against the authors in terms of interpreting their results, but also making it much more difficult for participants to keep track of face-gaze-valence associations. This limitation should be acknowledged, and care taken to avoid over-interpreting spurious results.*

**Response 17:** We thank the reviewer for this comment.

We agree with your idea and also consider that this possibility should be carefully discussed (p. 33, lines 528-529).

**Comment 18:**

*P.20. There were two ratings for each face that were then averaged. Please provide a measure of consistency or reliability across ratings. The authors replied to a comment from the reviewers in the previous round that said that these ratings were consistent, but I would like to see this in the manuscript (a footnote would suffice).*

**Response 18:** We thank the reviewer for this insightful comment. We have added these results to a footnote in the Results section (p. 24).

**Comment 19:**

*P.23. “Moreover, BF10 in the condition comparing positive and neutral valence-cue was 2.62, indicating that positive emotional valence of gaze at targets increased facial trustworthiness.” This Bayes factor indicates anecdotal evidence, and so the support for this interpretation is weak at best.*

**Comment 22:**

*P.25. All of the Bayes factors here indicate very weak evidence either way, and this analysis provides no conclusive answer to any of the contrasts described.*

**Response 19 and 22:** We thank the reviewer for this comment. To discuss this more carefully, we have changed the expression of the interpretation of these results (p. 26, 28-29).

**Comment 20:**

*P.25. “Because the non-predictive faces looked at the positive or the negative target with equal probability, we can assume that the perceived trustworthiness of the non-predictive faces were not systematically modulated by repetitive presentations in the gaze-cue phase.” Not necessarily. Learning about faces may be biased towards cheater detection and learning about invalid faces may be prioritised as a result (Bell et al., 2012; Buchner et al., 2009; Strachan & Tipper, 2017).*

**Response 20:** We thank the reviewer for this comment. We are sorry that our explanation was insufficient and has caused a misunderstanding. To clarify this, we have revised the prediction of the results (p. 28).

**Comment 21:**

*P.25. The data used for this analysis do not seem to be in the Supplementary Material.*

**Response 21:** We thank the reviewer for this comment. We have included all the data to Supplementary Materials.

**Comment 23:**

*P.27. “However, we cannot disregard the possibility that the emotional information in the images boosted trustworthiness or enhanced another positive affect such as attractiveness. In future research, we should examine the types of impressions that are influenced by the emotional valence of gazed images or events.” With regards to directionality, the continuous pre- and post-gaze-cueing continuous trust ratings introduced in Manssuer et al. (2015) allow for easier tracking of whether this learning effect is driven by an increase in trust for valid faces or a decrease in trust for invalid faces relative to baseline. This could be worth considering when thinking about future research.*

**Response 23:** We deeply appreciate the reviewer for this comment. We want to consider this point in future studies.

**Comment 24:**

*P.27. Line 441-446. This interpretation is based on very weak evidence and inconclusive Bayesian analysis. I do not feel that these conclusions are justified.*

**Response 24:** We thank the reviewer for this comment.

According to your comment, we have included a discussion about this point (p. 29 line 460-464).

**Comment 25:**

*A general point regarding the effect that the authors are investigating: since the original Bayliss & Tipper (2006) paper, there have been several studies from Steven Tipper’s lab that have replicated and extended the trust learning effect, and these form the bulk of the literature on this effect. I have mentioned a few in this review (including a couple of my own). However, one that I think is of particular interest and should be discussed is Kirkham et al. (2015) PLOS One. “Facial Mimicry and Emotion Consistency: Influences of Memory and Context.” This addresses a very similar question to the current study but uses overt expressions of emotion to manipulate trust and find that faces that show inconsistent expressions (i.e. smiling at negative targets or frowning at positive targets) are distrusted. Embedding the current study in the context of existing literature and interpreting the findings in light of previous work will greatly strengthen the theoretical contribution of the paper.*

**Response 25:** We highly appreciate the reviewer for this insightful comment. As you have stated, we have attempted to embed our findings in the literature of previous studies (such as Kirkham et al., 2015), and then explain the value of the present study. Thus, we have revised the General discussion.
