# Supplementary material for: Affective evaluation of images influences personality judgments through gaze perception
Source: PLoS One. 2020 Nov 5;15(11):e0241351. doi: 10.1371/journal.pone.0241351 (PMC7643958; doi:10.1371/journal.pone.0241351)
Supplement: Attachment — Submitted filename: Response to Reviewers.docx [file pone.0241351.s005.docx]

**RESPONSE TO REVIEWER 1:**

Reference: PONE-D-19-14867R3

Resubmission

“Affective evaluation of images influences personality judgments through gaze perception”

We highly appreciate you for taking the time to make valuable comments on the above entitled manuscript despite your busy schedule. We have revised the manuscript according to your comments. Our responses to each reviewers’ comments are listed below.

**Comment 1:** *change to lines 88-95: Upon first reading this section, I got the impression that the authors were trying to say that some research shows a link between perceived trustworthiness and gaze cueing, while others do not. If that is the case, then the Bayliss & Tipper (2006) paper should not be listed as evidence of the former, but rather of the latter. I think what I’m confused by is the use of “attentional facilitation” and “[magnitude] of [gaze] cueing effects”, and how/whether they differ. If the authors mean for them to be the same, then using consistent wording would get that across more clearly.*

*After looking at the references used to support the two sides, my second impression is that the authors are trying to delineate whether gaze preference leads to liking certain objects more, or liking (trusting) certain faces more? So the directionality between gaze conferring special status on objects, versus gaze behaviours ‘rebounding’ onto that individual to guide trustworthy judgments of said face.*

*In either case, I believe the authors need to be more explicit about what they mean here.*

*As an aside, I suggest using the term “magnitude” rather than “degree” when discussing modulations of the cueing effect throughout the text.*

**Response 1:** Thank you for this insightful comment.

As you have stated, this sentence was unclear. After considering all the reviewers’ comments comprehensively, we considered that the section on this correlation analysis might confuse the readers’ understanding of the main results of our study because the hypothesis of the correlation analysis was developed from a different perspective from the main body of this study.

Moreover, as you have pointed out, we also considered it difficult to draw clear conclusions and develop a meaningful discussion based only on this correlation analysis. Therefore, we removed the description of the correlation analysis from the revised manuscript. We believe that this change does not significantly influence our study’s primary results and hope that this change would contribute to a better understanding of our study.

**Comment 2:** *Figure 2 caption: this is much improved. I would suggest removing the word “pairs” from “there were 6 pairs of faces” and “there were 8 pairs of faces” – this reads as though you only had 12 faces in E1 and 16 faces in E2, which is not true.*

**Response 2:**

Thank you for this comment. We have removed the word “pairs” according to your comment.

**Comment 3:** *Gaze cueing in positive target condition: I would argue that the authors can only draw conclusions about the magnitude of the gaze cueing effect if the ANOVA contrasted just valid and invalid trials. While some of the early gaze cueing literature used a neutral condition to calculate costs and benefits (e.g., Friesen & Kingstone, 1998), this is no longer the norm; rather, a direct contrast between valid and invalid trials is conducted. If the authors run an ANOVA with face type (valid-cue, invalid-cue) and target type (negative, positive), do they see a main effect of face type that does not interact with target type. This analysis is more in-line with the vast majority of analyses in this field, thus I believe it would be warranted to include it. Otherwise, one could argue that the much slower RTs in the no-cue condition were driving the ‘cueing effect’ the authors claim is present.*

**Response 3:** We welcome this insightful comment. As you have stated, we also consider conducting an analysis that is similar to the vast majority if analyses in this field would help the readers better interpret our results. Therefore, we have included the following sentences in Lines 215-238, based on your comment, “*We conducted an ANOVA for the no-cue condition described above. However, nearly all gaze cueing studies examining the effect of gaze cues have directly contrasted response times between valid- and invalid-cue conditions. Therefore, we additionally conducted an ANOVA with face type (valid-cue and invalid-cue) and target type (negative and positive), similar to many previous studies. Results indicated that the main effect of face type (F(1, 19) = 8.01, p = .01, ηp2 = .30) and its interaction with target type were significant (F(1, 19) = 4.42, p = .05, ηp2 = .19). Multiple comparisons showed that the response times were faster for valid than for invalid cue trials when the target was negative (F(1, 19) = 11.29, p < .01, ηp2 = .37), which was not the case when the target type was positive (F(1, 19) = 1.75, p = .20, ηp2 = .08). Moreover, the response times for valid cue trials were faster when the target type was negative than when the target type was positive (F(1, 19) = 6.19, p < .05, ηp2 = .25). There were no significant differences in response times between positive and negative targets for invalid cue trials (F(1, 19) = 0.64, p = .43, ηp2 = .03). Moreover, the main effect of target type was not significant, F(1, 19) = 0.80, p = .38, ηp2 = .04.*”

**Comment 4:** *lines 220-222: the authors should reword this section slightly. “While numerically there appears to be a difference between the positive and negative targets, the two-way interaction between …”*

**Response 4:** Thank you for your comment. According to your comments, we have changed the description of this point (Lines 285-287) as follows, “*While numerically there appears to be a difference between the positive and negative targets, the two-way interaction between face type and target type was not significant, F(1, 19) = 3.41, p = .08, ηp2 = .15*.”

**Comment 5:** Bayes (lines 400-408): The authors should remove the writing between lines 400 and 405, beginning “Moreover, BF10...” and concluding “(BF10 = 1.88)”. The highlighted new section provides the Bayes analyses without overselling them or having the findings repeated twice.

**Response 5:** We thank the reviewer for this valuable comment. We removed these sentences in the revised manuscript according to your comment. Moreover, we changed the description of the Bayes analysis to exclude repetitions (Lines 514-525).

**RESPONSE TO REVIEWER 2:**

Reference: PONE-D-19-14867R3

Resubmission

“Affective evaluation of images influences personality judgments through gaze perception”

We highly appreciate you for taking the time to make valuable comments on the above entitled manuscript despite your busy schedule. We have revised the manuscript according to your comments. Our responses to each reviewers’ comments are listed below.

**Comment 1:** *The only point I am unsure about (which I did not notice in the last revision) is the power calculation for Experiment 1 where the authors use an intraclass correlation coefficient of zero. As far as I understand, the ICC in this case refers to the similarity between values from the same participant. An ICC of zero therefore predicts no individual variation in the sample (e.g. that individuals will not give overall higher ratings or overall lower ratings than others ). This does not seem a plausible prediction to me, but as I do not have the statistical expertise to say how such an assumption affects the sample size estimate I will leave this to the authors and AE.*

**Response 1:** Thank you for this insightful comment. We agree with your point. There is some debate on ICC settings in situations in which the ICC cannot be known in advance, and there are recommendations for setting it to 0.5 or 0. In this study, we used 0. We believe that the ICC setting did not significantly influence the results of our study.

**Comment 2:**  *“We predicted that the emotional valence of images would be more transferred to the gazer’s personality when the sender’s gaze direction matched the location of emotional images” – This is unclear, and ‘more transferred’ is an odd way of phrasing. What is wrong with just saying, “emotional valence of images would affect personality judgements only when the face looked toward the image rather than away from it”?*

**Response 2:** Thank you for your insightful comments. Based on your comment, we revised this sentence in the revised manuscript as follows, “*We predicted that the emotional valence of images would affect the personality judgments only when the face looked toward the image rather than away from it.*” (in lines 83-86)

**Comment 3:** *Once again, I caution the authors to be mindful of confusing likeability and trustworthiness (e.g. p.6 Lines 91-93)*

**Response 3:** We thank the reviewer for this comment. We have revised manuscript with attention to the use of words, likability and trustworthiness.

**Comment 4:** *P.9-10 Lines 146-156: This covers predictions about the third cueing condition that should have been spelt out in the introduction*

**Response 4:** We thank you for your insightful comments. We have moved these sentences to the introduction of the revised manuscript according to your comment (Lines 90-98).

**Comment 5:** *Change the labels on Figs 1 and 2, as currently Figure 2 appears first in the manuscript.*

**Response 5:** We thank you for your insightful comment. We changed the labels on Figures 1 and 2 of the revised manuscript.

**Comment 6:** *P.14 line 231-232: “A BF10 greater than 3.0 provides moderate support for the null hypothesis” – This is incorrect. This should be either “BF01”, or “alternative hypothesis”*

**Comment 7:** *P.14 line 233-235: The authors have just explained how BF10 works, so there is no need to repeat this in such confusing terms. Spell out how the predictions will translate to results in clear, simple terms (e.g. “Under the hypothesis that the valence of gazed images affects facial evaluation, we expected to find strong evidence (BF10 > 10) of a difference in ratings of valid faces between positive and negative targets.”)*

**Comment 8:** *P.15 line 242: Similarly, “This finding suggests the validity of models supporting the hypothesis that…” is overly complicated and difficult to parse. It is much simpler to just say, “This finding supports the hypothesis that…” It is worth going through the Results section (the sections with Bayesian analysis in particular) and simplifying the sentences wherever possible to achieve maximum clarity.*

**Comment 12:** *P.27: “The BF10 supporting the hypothesis that… was slightly higher than BF01, which supported the null” – Again, this is unclear because it suggests that the results support the null when in fact it is the opposite. Use “support” exclusively to refer to the current results and simplify the description of the results overall to avoid such misinterpretations. Also, bear in mind that there is little need to report both BF01 and BF10 at the same time – these values are the inverse of each other, and so if BF10 is >1 then that is evidence supporting the alternative, and if it is <1 then that is evidence supporting the null (although it may not be worth interpreting if it is so close to 1). Just reporting one of these may help to simplify the reporting of these tests substantially.*

**Response 6, 7, 8, and 12:** We thank you for your insightful comment. It is correct that *“A BF10 greater than 3.0 provides moderate support for the null hypothesis”*. We have fixed these mistakes. Moreover, according to your comments, we also simplified the reporting of BF results to make it easier to understand (Lines 301-311, 518-531, 547-555).

**Comment 9:** *P.20: Why is the result of the power analysis different in Experiment 2 if it used the same criteria as that of Experiment 1? If the criteria were different (i.e. the effect size was changed to match an observed effect size from Experiment 1) then this needs to be reported.*

**Response 9:** We thank you for your insightful comment. The results of the power analysis were different because the factorial design was different between Experiment 1 (3 × 2 factorial design) and Experiment 2 (2 × 4 factorial design).

**Comment 10:** *P.23 line 378: “target position cues” should read “valence-target and position cues”, no?*

**Response 10:** We thank you for your insightful comment. You are right. We have changed this sentence to “valence-target and position cues” (Line 507).

**Comment 11:** *P.23 footnote: “differences […] were not significant and that in both cases the faces gaze toward the negative [t] and positive images, [t]” – The wording of this suggests that these stats tested which way the faces were looking, rather than changes in evaluations to different types of faces. I suggest changing this to, “differences […] were not significant, both in cases where…”*

**Response 11:** We thank the reviewer for this insightful comment. We changed this sentence in the revised manuscript based on your comment as follows, “*The results showed that differences between the number of evaluations were neither significant in cases in which faces gazed toward negative, t(45.27) = -0.49, p = .62, nor positive images, t(45.94) = 0.11, p = .92, suggesting that the number of evaluations did not affect facial evaluations in Experiment 2.*” (P. 22 footnote)

**Comment 13:** *P.29 line 473: “to clarify whether the emotional valence of a gaze target affected the impression of the gazers’.” should read, “gazer’s trustworthiness” or something similar.*

**Response 13:** We thank the reviewer for this comment. We changed this sentence in the revised manuscript according to your comment as follows, “*The present study was designed to clarify whether the emotional valence of a gazed target affected the gazer’s trustworthiness*”. (Line 574)
