# Supplementary material for: Affective evaluation of images influences personality judgments through gaze perception
Source: PLoS One. 2020 Nov 5;15(11):e0241351. doi: 10.1371/journal.pone.0241351 (PMC7643958; doi:10.1371/journal.pone.0241351)
Supplement: Attachment — Submitted filename: Response to Reviewers.docx [file pone.0241351.s006.docx]

**RESPONSE TO REVIEWER 1:**

Reference: PONE-D-19-14867R4

Resubmission

“Affective evaluation of images influences personality judgments through gaze perception”

We highly appreciate you for taking the time off your busy schedule to make valuable comments on the above entitled manuscript. We have revised the manuscript according to your comments. Our responses to each reviewers’ comments are listed below.

**Comment 1:** *change to lines 88-95: this reads much better! One tiny change I would suggest is to use the term “target” instead of “image”: We predicted that the emotional valence of images would affect personality judgments only when the face looked toward the TARGET rather than away from it.” – the authors should make this change in the subsequent sentence as well, for clarity.*

**Response 1:** We have changed from “image” to “target,” according to your comment (line 85).

**Comment 2:** *Figure captions: I believe the authors swapped figures 1 and 2, however didn’t rename the corresponding figure captions. Further, while they took out the word “pairs”, the did not take out the word “of” that came after the word “pairs”. Please proofread this caption to remove the errors.*

**Response 2:** Thank you for this comment.

We carefully proofread the manuscript and removed the word “of ” according to your comment (lines 168, 169).

**Comment 3:** *Additional ANOVA: Thank you for running the additional analysis. Please provide a few references to support the sentence stating that “nearly all gaze cueing studies…invalid-cue conditions.”*

**Response 3:** We thank the reviewer for this comment.

We have provided references to support this sentence (line 217).

**RESPONSE TO REVIEWER 3:**

Reference: PONE-D-19-14867R4

Resubmission

“Affective evaluation of images influences personality judgments through gaze perception”

We highly appreciate you for taking the time off your busy schedule to make valuable comments on the above-entitled manuscript. We have revised the manuscript according to your comments. Our responses to each reviewers’ comments are listed below.

**Comment 1:** *I think that the decision to drop the correlation analysis from the main text was a good one as it was clearly motivated by different hypotheses than the authors set out to test, although I think it may be preferable to report this analysis in the Supplemental Material than to leave it out completely (particularly as the data are still included in the S2 Appendix).*

**Response 1:** Thank you for this insightful comment. We considered that the correlation analysis did not provide the readers with a clear and specific conclusion. Therefore, we decided to remove information about the correlation analysis from the Supplemental Material and S2 Appendix.

**Comment 2:** *It is possible that I overlooked a value or have misunderstood something that the authors know about the logic of G*Power, but I think it is also possible that the sample size criteria in this study were based on erroneous calculations. For example, G*Power is unable to handle designs with more than one within-subject factor (see https://www.researchgate.net/post/How_compute_a_repeated_measure_power_analysis_in_Gpower for a description and the workaround solution by David Morse that I used when trying to replicate the analysis). The only way to know is to allow readers to fully replicate the power analyses.*

*I have listed the G*Power settings that should be reported, with the values I used for my attempt at replication based on the 2x3 within-subjects interaction in Experiment 1 (ANOVA: Repeated measures, within factors; f=0.25; alpha=0.05; 1-beta=0.80; no. groups=1; no. measurements=3; corr. among rep measures=0.0; nonsphericity correction=1 – estimated sample size: 53).*

*I also advise the authors not to use G*Power again to estimate power for future studies using a within-subjects factorial design. Simulation-based approaches (such as using the Superpower app: http://shiny.ieis.tue.nl/anova_power) are generally better suited for this.*

**Response 2:** Thank you for your insightful comments.

As you have stated, we found that the G*Power settings in this study might be odd.

We have listed the G*Power settings used in Experiment 1 of our study (ANOVA: Repeated measures, within factors; f = 0.25; alpha = 0.05; 1-beta = 0.80; number of groups = 1; number of measurements = 6; corr. among rep measures = 0.0; nonsphericity correction = 0.2 (1/ (number of measurements-1)); estimated sample size: 107/6 (number of measurements) = 17.83).

Moreover, using G*power may not have been the best choice among the various power analysis methods. We highly appreciate the reviewer for providing information on simulation-based power analysis approaches. We would like to use this method in the future studies. We calculated the power in our study by using the power analysis that you recommended. As a result, the power of the 2x3 within-subjects interactions was 86.42 % when the sample size was 20.

Therefore, we believe that the power was not too small.

Since the current study used the G*power for the sample size calculation, we believe that information about the G*power should be included in this paper.

Thus, we have added and rewritten the explanation of G*power's analysis to avoid providing misleading information to readers based on your comments as follows “*We conducted an advance power analysis (G*power; [16, 17]) in to determine the minimum sample size by adopting the following settings: a medium effect size (f) of 0.25, a significance level of 5%, 1 group, 6 measurements, a correction for nonsphericity of 0.2, and an intra-class correlation coefficient of zero. It was decided that a minimum of 18 participants was needed to achieve a power level of 0.80.*” (lines 103-106, 325-326).

**Comment 3:** *p.5, line 70 ‘module’ should be ‘modulate’*

**Response 3:** We thank the reviewer for this comment. We have changed ‘module’ to ‘modulate’ (line 70).

**Comment 4:** *The figure legends in the text are still incorrect – ‘Fig 2’ on p.9 is the first figure, for example, and the legend refers to the figure that has been uploaded as Figure 2, while Figure 1 does not appear until p.11. It is not enough to swap the labels around – the authors should take care that the figures are presented, labelled, and referred to appropriately throughout the document.*

**Response 4:** We apologize for making you point out this mistake repeatedly. We thank you for your repeated correction. We have carefully revised and changed the legends of the Figures according to your comment.

**Comment 5:** *p.23, lines 378-381: “Results showed that the BF10, in the comparison between positive and negative valence-target conditions, was 6.82, which moderately supports the alternative hypothesis. This result supported our contention that faces always looking at positive images are evaluated as more trustworthy than faces always looking at negative images.” – Specify that this contrast is for valid faces (i.e. “the comparison of ratings for valid faces…”)*

**Response 5:** We thank you for your insightful comment. We have added an explanation about the condition to clarify this point as follows, “*Results indicated that BF10 for comparing positive and negative valence-target conditions for valid faces was 6.82, which moderately supported the alternative hypothesis.*” (lines 396-397).

**Comment 6:** *p.25 The additional analysis is not reproducible using the data available in the Supplemental Material. This requires at least having the ratings for valid-random faces broken down by the target they looked at on the previous trial.*

**Response 6:** We thank you for your insightful comment. We included data regarding the additional analysis in the Supplemental Material, according to your comment.
